# Impact of complexity in minimally invasive liver surgery on enhanced recovery measures: prospective study

**DOI:** 10.1093/bjsopen/zrad147

**Published:** 2024-01-19

**Authors:** Paul M Dahlke, Christian Benzing, Georg Lurje, Thomas Malinka, Nathanael Raschzok, Can Kamali, Safak Gül-Klein, Wenzel Schöning, Karl H Hillebrandt, Johann Pratschke, Jens Neudecker, Felix Krenzien

**Affiliations:** Department of Surgery, Charité–Universitätsmedizin, Corporate Member of Freie Universität Berlin, Humboldt-Universität zu Berlin, Campus Charité Mitte and Campus Virchow-Klinikum, Berlin, Germany; Department of Surgery, Charité–Universitätsmedizin, Corporate Member of Freie Universität Berlin, Humboldt-Universität zu Berlin, Campus Charité Mitte and Campus Virchow-Klinikum, Berlin, Germany; Department of Surgery, Charité–Universitätsmedizin, Corporate Member of Freie Universität Berlin, Humboldt-Universität zu Berlin, Campus Charité Mitte and Campus Virchow-Klinikum, Berlin, Germany; Department of Surgery, Charité–Universitätsmedizin, Corporate Member of Freie Universität Berlin, Humboldt-Universität zu Berlin, Campus Charité Mitte and Campus Virchow-Klinikum, Berlin, Germany; Department of Surgery, Charité–Universitätsmedizin, Corporate Member of Freie Universität Berlin, Humboldt-Universität zu Berlin, Campus Charité Mitte and Campus Virchow-Klinikum, Berlin, Germany; Clinician Scientist Program, Berlin Institute of Health (BIH), Anna-Louisa-Karsch-Str. 2, 10178, Berlin, Germany; Department of Surgery, Charité–Universitätsmedizin, Corporate Member of Freie Universität Berlin, Humboldt-Universität zu Berlin, Campus Charité Mitte and Campus Virchow-Klinikum, Berlin, Germany; Department of Surgery, Charité–Universitätsmedizin, Corporate Member of Freie Universität Berlin, Humboldt-Universität zu Berlin, Campus Charité Mitte and Campus Virchow-Klinikum, Berlin, Germany; Department of Surgery, Charité–Universitätsmedizin, Corporate Member of Freie Universität Berlin, Humboldt-Universität zu Berlin, Campus Charité Mitte and Campus Virchow-Klinikum, Berlin, Germany; Department of Surgery, Charité–Universitätsmedizin, Corporate Member of Freie Universität Berlin, Humboldt-Universität zu Berlin, Campus Charité Mitte and Campus Virchow-Klinikum, Berlin, Germany; Clinician Scientist Program, Berlin Institute of Health (BIH), Anna-Louisa-Karsch-Str. 2, 10178, Berlin, Germany; Department of Surgery, Charité–Universitätsmedizin, Corporate Member of Freie Universität Berlin, Humboldt-Universität zu Berlin, Campus Charité Mitte and Campus Virchow-Klinikum, Berlin, Germany; Department of Surgery, Charité–Universitätsmedizin, Corporate Member of Freie Universität Berlin, Humboldt-Universität zu Berlin, Campus Charité Mitte and Campus Virchow-Klinikum, Berlin, Germany; Department of Surgery, Charité–Universitätsmedizin, Corporate Member of Freie Universität Berlin, Humboldt-Universität zu Berlin, Campus Charité Mitte and Campus Virchow-Klinikum, Berlin, Germany; Clinician Scientist Program, Berlin Institute of Health (BIH), Anna-Louisa-Karsch-Str. 2, 10178, Berlin, Germany

## Abstract

**Background:**

Adherence to enhanced recovery after surgery (ERAS) protocols is crucial for successful liver surgery. The aim of this study was to assess the impact of minimally invasive liver surgery complexity on adherence after implementing an ERAS protocol.

**Methods:**

Between July 2018 and August 2021, a prospective observational study involving minimally invasive liver surgery patients was conducted. Perioperative treatment followed ERAS guidelines and was recorded in the ERAS interactive audit system. Kruskal–Wallis and ANOVA tests were used for analysis, and pairwise comparisons utilized Wilcoxon rank sum and Welch's *t*-tests, adjusted using Bonferroni correction.

**Results:**

A total of 243 patients were enrolled and categorized into four groups based on the Iwate criteria: low (*n* = 17), intermediate (*n* = 81), advanced (*n* = 74) and expert difficulty (*n* = 71). Complexity correlated with increased overall and major morbidity rate, as well as longer length of stay (all *P* < 0.001; standardized mean difference = 0.036, 0.451, 0.543 respectively). Adherence to ERAS measures decreased with higher complexity (*P* < 0.001). Overall adherence was 65.4%. Medical staff-centred adherence was 79.9%, while patient-centred adherence was 38.9% (*P* < 0.001). Complexity significantly affected patient-centred adherence (*P* < 0.001; standardized mean difference (SMD) = 0.420), but not medical staff-centred adherence (*P* = 0.098; SMD = 0.315). Postoperative phase adherence showed major differences among complexity groups (*P* < 0.001, SMD = 0.376), with mobilization measures adhered to less in higher complexity cases.

**Conclusion:**

The complexity of minimally invasive liver surgery procedures impacts ERAS protocol adherence for each patient. This can be addressed using complexity-adjusted cut-offs and ‘gradual adherence’ based on the relative proportion of cut-off values achieved.

## Teaser

Adherence is essential as a key assessment parameter establishing an Enhanced Recovery After Surgery protocol in liver surgery. Overall adherence to an Enhanced Recovery After Surgery protocol decreases with increasing complexity of a minimally invasive liver surgery procedure. Furthermore, patient-centred adherence is significantly lower than medical staff-centred adherence.

**Figure:**
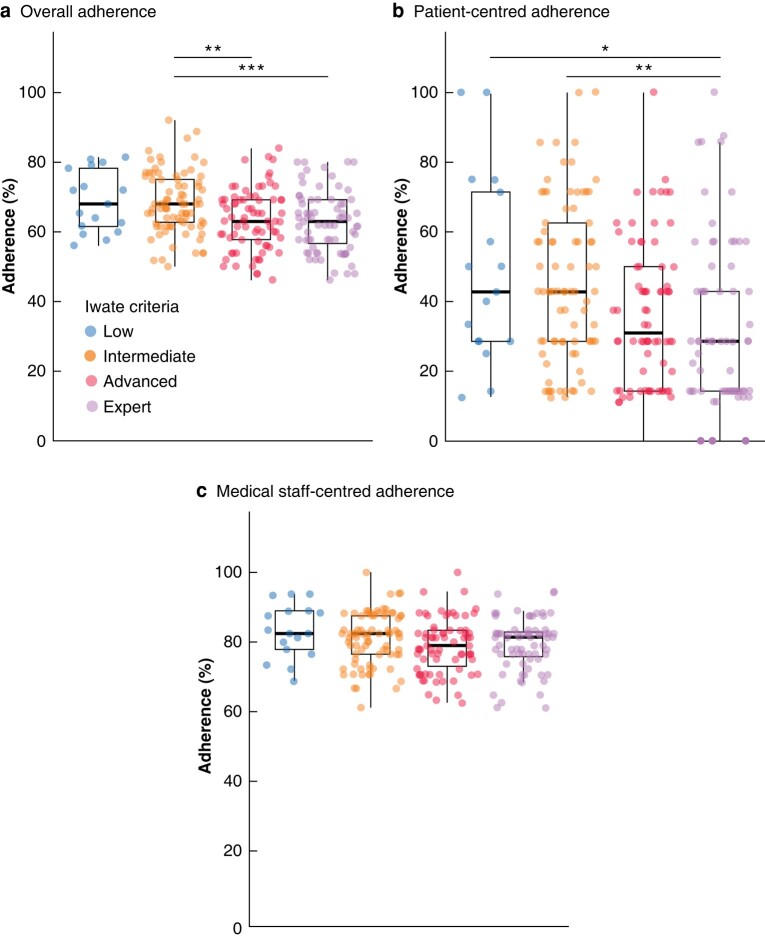

## Introduction

Perioperative concepts like fast-track or enhanced recovery after surgery (ERAS) have been introduced to liver surgery to improve outcomes and patient care^1,2^. In 2016, the first ERAS guideline for liver surgery from the ERAS Society was published and then updated in 2022^3,4^. A total of 26 ERAS items are claimed by the ERAS Society, with adherence defined by how well each of these items is performed.

Adherence as an assessment parameter is not intended to improve outcome per se but to monitor the implementation and performance of an ERAS programme. Surprisingly, the adherence to individual ERAS measures is neglected in most studies of liver surgery. In a large recent meta-analysis on the implementation of ERAS protocols in liver surgery by Noba *et al*., only four of 27 studies (474 of 3739 patients) reported on adherence to their ERAS protocol, ranging from 65% to 92.2%^5–7^. The high variance in adherence may be due in part to the fact that different ERAS protocols were used in each study, resulting in individual adherence calculations. For example, the adherence measures for mobilization range from a vague ‘mobilization should be performed within the first 24 hours’ to more precise specifications such as ‘mobilization should be performed more than 4 hours on postoperative day (POD)1’. Those thresholds were defined individually in each protocol rendering the adherence data barely comparable^5–7^. On the other hand, the proportions of patients undergoing minimally invasive surgery differed greatly between the studies (24.7% for Labgaa *et al*., 40% for Teixeira *et al*., 0% for Jones *et al*.)^6,8,9^. It is difficult to draw any conclusion about the applicability of the respective ERAS measures when minimally invasive surgery and open surgery are subsumed with well proven differences in complication rate and outcome^10,11^.

Laparoscopic and robotic liver resections have a high variance in complexity ranging from atypical segment III liver resections to extended hemihepatectomy (trisectorectomy)^12,13^. Hence, the clinical course of patients can be highly variable with high variance in the frequencies of postoperative complications, patient management and length of stay. That is why ERAS concepts and adherence rates as a one-size-fits-all concept are questionable. This has not been addressed by recent studies nor are cut-off values of adherence protocols adjusted according to the complexity of the liver surgery.

The aim of this study was to evaluate the impact of the complexity of minimally invasive liver surgery (MILS) on the applicability and adherence of perioperative ERAS measures.

## Methods

This prospective study was approved by the local ethics committee under application numbers EA2/108/18 and EA4/153/18 and was registered with the German Clinical Trials Register (DRKS00030908). From July 2018 to August 2021, patients undergoing elective liver resection within an ERAS programme at the Department of Surgery, Campus Virchow-Klinikum, Charité–Universitätsmedizin Berlin, were included. The perioperative treatment was based on the ERAS protocol of the ERAS Interactive Audit System (EIAS) (Encare, Stockholm, Sweden)^2^. Data on perioperative adherence and complications were recorded. STROBE statements of reporting observational studies were taken into consideration (STROBE Checklist)^14^.

### Inclusion and exclusion criteria

Patients who were at least 18 years old and underwent elective MILS using the ERAS protocol were included. Written informed consent had to be obtained from the patient prior to treatment.

Patients who underwent open liver surgery were excluded. In addition, patients were excluded if the procedure started laparoscopically but was converted to open or no liver resection was performed (for example for peritoneal carcinosis) or if a synchronous resection of another organ was performed (for example simultaneous colorectal resection).

### ERAS protocol and adherence calculation

The ERAS protocol was implemented in 2019 and supervised by an ERAS core team, consisting of surgeons, anaesthetists, physiotherapists, nursing staff and ERAS nurses. Based on the ERAS measures of the EIAS, standard operating procedures (ERAS protocol, *Table S1*) as well as patient information brochures and patient diaries were created. The implementation of the ERAS protocol comprised specific interdisciplinary ERAS training of the staff and regular audit meetings.

The applied ERAS protocol was based on the ERAS guidelines for liver surgery^3^. The individual ERAS measures applied, and their corresponding adherence definitions are listed in *Table S1*.

Adherence was grouped into preadmission, preoperative, intraoperative and postoperative phase. Adherence was classified in patient-centred ERAS measures, where the patient was responsible for adherence and medical staff-centred ERAS measures, where the medical staff were responsible for adherence (*Table 1*). If a measure was not applicable or not recorded, it was not included in the calculation for this specific patient.

**Table 1:** ERAS protocol measures and responsibilities

Medical staff-centred ERAS measures (ERAS–nurse, surgeon, anaesthetist, physiotherapist)		Patient-centred ERAS measures	
**Preadmission phase**			
1	Nutritional status	3	Smoking behaviour (cessation)
2	Preoperative immunonutrition	4	Alcohol consumption (cessation)
**Preoperative phase**			
5	Education on the ERAS programme	6	Carb loading
7	Bowel preparation		
8	Preoperative sedative medication		
9	Antibiotic prophylaxis		
10	Thrombosis prophylaxis		
11	Steroid administration		
**Intraoperative phase**			
12	Type of incision		
13	Abdominal drains		
14	Omentoplasty		
15	PONV prophylaxis		
16	Systemic opioid administration		
17	Epidural anaesthesia		
18	Upper body warming		
19	Use of 0.9% NaCl		
20	Removal of gastric tube		
21	Central venous pressure		
**Postoperative phase**			
22	Termination of i.v. fluid administration	24	Energy consumption at POD0
23	Postoperative weight gain	25	Energy consumption at POD1
30	Removal of IUC	26	Mobilization at all on the day of surgery
31	Control postoperative glycaemia	27	Mobilization on POD1
32	Postoperative epidural analgesia	28	Mobilization on POD2
33	30-day follow-up	29	Mobilization on POD3

ERAS measures are divided according to whether adherence fulfilment is more dependent on the medical staff or on the patient. Adherence conditions for each ERAS measure can be found in *Table S1*. ERAS, enhanced recovery after surgery; IUC, indwelling urinary catheter; i.v., intravenous; NaCl, sodium chloride; POD, postoperative day; PONV, postoperative nausea and vomiting.

### Definition of complexity

A preoperative distinction of difficulty of MILS was made by classifying the procedures according to the Iwate criteria (IC), which include tumour location and size, extent of liver resection, proximity to major vessels, use of hand-assisted laparoscopic surgery and the preoperative Child Pugh class^15^. Liver resections are classified as low (1–3 IC points), intermediate (4–6 IC points), advanced (7–9 IC points) and expert level (10–12 IC points)^12,16,17^.

### Statistics

Statistical analyses were performed using R (version 4.1.2; R Foundation for Statistical Computing, Vienna, Austria).

Analysis between the four groups was performed using Kruskal–Wallis for categorical variables and using ANOVA for quantitative variables. For pairwise comparison between groups, a pairwise Wilcoxon rank sum test was chosen for categorical variables and a pairwise Welch´s *t*-test for quantitative variables with Bonferroni adjustment. Bonferroni-adjusted *P* values for pairwise comparisons are marked as ‘P_adj_’. The significance level (α-level) chosen was 0.05.

## Results

### Patient characteristics

Between July 2018 and August 2021, 243 patients who underwent MILS were prospectively enrolled (*Fig. S1*). According to the before surgery determined IC, the difficulty of the procedures was classified as low in 17 patients (7.0%), intermediate in 81 patients (33.3%), advanced in 74 patients (30.5%) and expert in 71 patients (29.2%).

### Clinical characteristics

Age, BMI, sex, and the distribution of patients with diabetes mellitus type II, or who smoked or consumed alcohol regularly before surgery, were comparable among the four groups (all *P* > 0.050; standardized mean difference (SMD) = 0.031, 0.125, 0.204, 0.072, 0.206, 0.219 respectively; *Table 2*). WHO performance status and ASA classification showed no significant differences between the groups (both *P* > 0.050; SMD = 0.251, 0.139 respectively; *Table 2*). The numbers of patients who had already undergone surgery in the right upper abdomen or received neoadjuvant radiotherapy were also comparable between groups (both *P* > 0.050; SMD = 0.095, 0.198 respectively; *Table 2*), while the number of patients receiving neoadjuvant chemotherapy was lowest in the low complexity group and highest in the expert complexity group (*n* = 2 (11.8%) in the low *versus n* = 30 (42.3%) in the expert group, *P* = 0.044; SMD = 0.380). After Bonferroni adjustment no significant differences between IC groups were observed (all *P*_adj_ > 0.050). This is also reflected by the higher number of patients with secondary malignant underlying liver disease in the groups with higher procedure complexity (*n* = 5 (29.4%) in the low *versus n* = 34 (47.9%) in the expert group, *P* = 0.017; SMD = 0.494), with no significant differences between the IC groups after Bonferroni adjustment.

**Table 2:** Baseline characteristics of the study cohort grouped by the Iwate criteria

Parameter	Low (*n* = 17)	Intermediate (*n* = 81)	Advanced (*n* = 74)	Expert (*n* = 71)	SMD	*P*
**Sex**					0.204	0.536
Male	6	43	40	35		
Female	11	38	34	36		
Age (years), mean(s.d.)	62.5(12.6)	62.5(12.1)	62.6(14.8)	61.8(13.6)	0.031	0.983
**BMI (kg/m^2^), mean(s.d.)**	25.4(3.6)	25.9(5.0)	26.1(4.8)	26.5(4.5)	0.125	0.824
<18.5	1 (5.9)	0 (0.0)	4 (5.4)	0 (0.0)	0.355	0.173
18.5–<25	7 (41.2)	39 (48.1)	28 (37.8)	30 (42.3)		
25–<30	7 (41.2)	27 (33.3)	29 (39.2)	22 (31.0)		
≥30	2 (11.8)	15 (18.5)	13 (17.6)	19 (26.8)		
Neoadjuvant chemotherapy	2 (11.8)	25 (30.9)	19 (25.7)	30 (42.3)	0.380	**0**.**044**
Neoadjuvant radiotherapy	0 (0.0)	2 (2.5)	3 (4.1)	4 (5.6)	0.198	0.622
History of surgery (right upper abdomen)	6 (35.3)	31 (38.3)	32 (43.2)	30 (42.2)	0.095	0.880
Smoked daily before surgery	2 (11.8)	14 (17.3)	17 (23.0)	7 (9.9)	0.206	0.184
Daily >3 standard glasses of alcohol before surgery	3 (17.6)	9 (11.1)	16 (21.6)	6 (8.5)	0.219	0.105
**Diabetes mellitus**	3 (17.6)	13 (16.0)	12 (16.2)	15 (21.1)	0.072	0.841
No diabetes mellitus	14 (82.4)	68 (84.0)	62 (83.8)	56 (78.9)	0.195	0.888
On diet control	3 (17.6)	12 (14.8)	10 (13.5)	12 (16.9)		
On medication	0 (0.0)	1 (1.2)	2 (2.7)	3 (4.2)		
**WHO-Performance Status**						
Asymptomatic	14 (82.4)	77 (95.1)	64 (86.5)	67 (94.4)	0.251	0.103
Symptomatic, ambulant	3 (17.6)	4 (4.9)	10 (13.5)	4 (5.6)		
Preoperative serum bilirubin (mmol/l), median (i.q.r.)	4.8 (2.2–10.3)	5.1 (0.6–7.5)	5.8 (3.4–8.825)	5.3 (2.55–8.2)	0.168	0.370
Preoperative serum albumin (g/l), median (i.q.r.)	43.2 (41.9–45.7)	42.8 (40.95–45.3)	44.05 (42.425–45.8)	42.15 (39.925–44.6)	0.262	**0**.**020**
**ASA classification**						
ASA I–II	9 (52.9)	33 (40.7)	39 (52.7)	34 (47.9)	0.139	0.477
ASA III–IV	8 (47.1)	48 (59.3)	35 (47.3)	37 (52.1)		
**Liver disease**						
Benign liver lesion	9 (52.9)	23 (28.4)	11 (14.9)	11 (15.5)	0.494	**0**.**017**
Primary liver carcinoma	3 (17.6)	23 (28.4)	26 (35.1)	26 (36.6)		
Secondary liver carcinoma	5 (29.4)	35 (43.2)	37 (50.0)	34 (47.9)		
**Type of surgery**						
Left hemihepatectomy	0 (0.0)	20 (24.7)	13 (17.6)	6 (8.5)	1.396	**<0**.**001**
Extended left hemihepatectomy	0 (0.0)	0 (0.0)	1 (1.4)	6 (8.5)		
Right hemihepatectomy	0 (0.0)	3 (3.7)	13 (17.6)	22 (31.0)		
Extended right hemihepatectomy	0 (0.0)	1 (1.2)	1 (1.4)	12 (16.9)		
Sectionectomy, segmentectomy	9 (52.9)	41 (50.6)	46 (62.2)	25 (35.2)		
Wedge or minor resection	8 (47.1)	16 (19.8)	0 (0.0)	0 (0.0)		
Operative time (min), median (i.q.r.)	117 (64–145)	160 (114–247)	251 (184–293)	282 (228–353)	1.156	**<0**.**001**
**Morbidity rate**	2 (11.8)	5 (6.2)	11 (14.9)	19 (26.8)	0.306	**0**.**006**
Clavien–Dindo I–II	2 (11.8)	3 (3.7)	5 (6.8)	3 (4.2)	0.172	0.516
Clavien–Dindo III–V	0 (0.0)	2 (2.5)	6 (8.1)	16 (22.5)	0.451	**<0**.**001**
Length of stay (days), median (i.q.r.)	5 (3–5)	5 (5–6)	6 (5–7)	7 (5–9)	0.543	**<0**.**001**

Values are *n* (%) unless otherwise indicated. Chi^2^ test, exact Fisher test or Kruskal–Wallis for categorical variables, ANOVA for continuous variables. INR, international normalized ratio; SMD, standardized mean difference; i.q.r., interquartile range. Statistically significant *P*-values are highlighted in bold.

### Surgical characteristics

There were significant differences in the types of resection that were performed between the four difficulty levels defined by the IC (*P* < 0.001; SMD = 1.396). While only sectionectomies or minor resections were performed in the low complexity group, hemihepatectomies and extended hemihepatectomies accounted for the major proportion of the more complicated procedures. Consequently, the operating time increased as the complexity of the procedure increased (117 (64–145) min in the low group *versus* 282 (228–353) min in the expert group, *P* < 0.001; SMD = 1.156; *Table 2*). After Bonferroni adjustment no significant differences in operating time between IC groups were observed (all *P*_adj_ > 0.050).

### Outcomes

Overall morbidity rate, Clavien–Dindo grade I–V^18^, showed highly significant differences (*n* = 2 (11.8%) in the low *versus*  *n* = 5 (6.2%) in the intermediate *versus*  *n* = 11 (14.9%) in the advanced *versus*  *n* = 19 (26.8%) in the expert group, *P* = 0.006; SMD = 0.306; *Table 2*). After Bonferroni adjustment, differences were found between the intermediate and expert groups (*P*_adj_ = 0.003). With rising complexity highly significant increases were also observed for major complications, Clavien–Dindo grade III–V (*n* = 0 (0.0%) in the low *versus*  *n* = 2 (2.5%) in the intermediate *versus*  *n* = 6 (8.1%) in the advanced *versus*  *n* = 16 (22.5%) in the expert group, *P* < 0.001; SMD = 0.451), with differences between the intermediate and expert groups as well, after adjusting for multiple testing (*P*_adj_ <0.001). Minor complications, Clavien–Dindo I–II, showed no differences between the IC groups. The length of stay increased with higher complexity (5 (3–5) days in the low *versus* 5 (5–6) days in the intermediate *versus* 6 (5–7) days in the advanced *versus* 7 (5–9) days in the expert group, *P* < 0.001; SMD = 0.543). Between the low and expert as well as intermediate and expert groups, these differences reached the level of significance after Bonferroni adjustment for multiple testing (*P*_adj_ = 0.034, *P*_adj_ = 0.002 respectively).

### Adherence characteristics

**Fig. 1:**
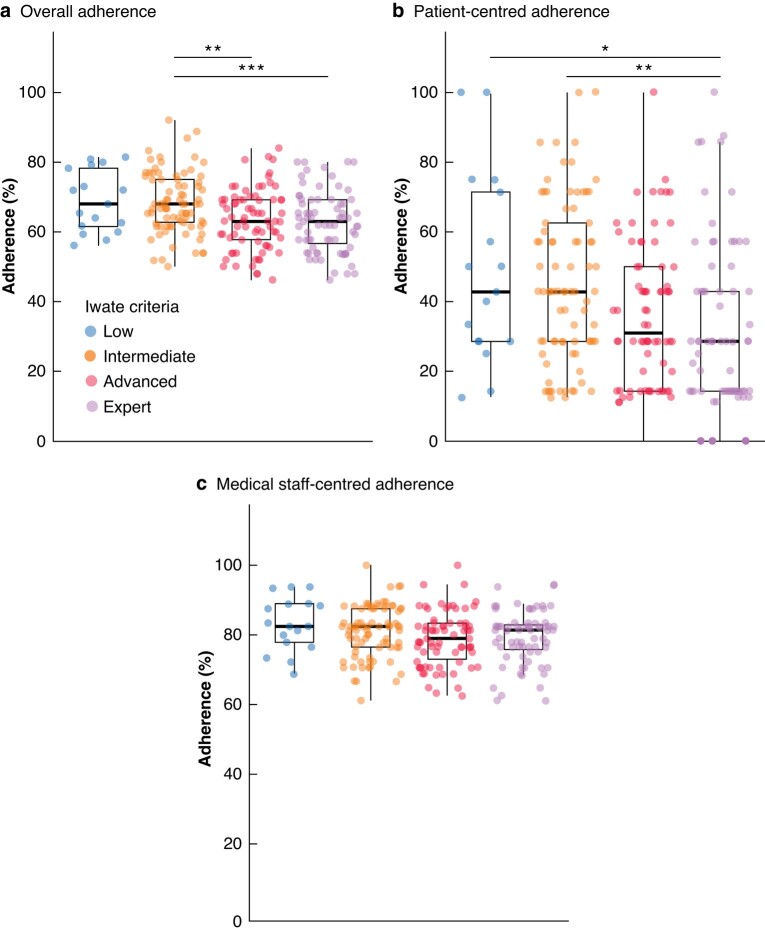
Total adherence (a), patient-centred adherence (b), and medical staff-centred adherence (c); the *y*-axis shows the adherence of patients defined according to the ERAS interactive audit system database The complexity of liver resection was graded according to the Iwate criteria. *P_adj_ < 0.05, **P_adj_ < 0.01, ***P_adj_ < 0.001, Welch´s *t*-test with Bonferroni correction.

**Fig. 2:**
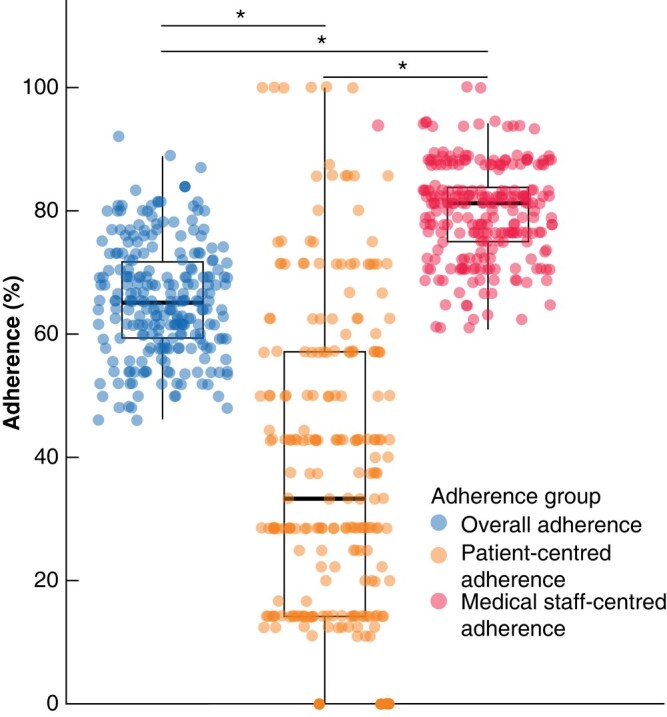
Comparison of overall adherence *versus* patient-centred adherence *versus* medical staff-centred adherence *P_adj_ < 0.0001, Welch´s *t*-test with Bonferroni correction.

The overall adherence to the ERAS protocol differed significantly between the IC groups (*Table 3*, *Figs. 1*, *2*, *Fig. S2*). Adherence decreased with increasing complexity of the liver resection (68.9% in the low *versus* 68.4% in the intermediate *versus* 63.6% in the advanced *versus* 62.9% in the expert group, *P* < 0.001; SMD = 0.432; *Fig. 1a*) with a mean overall adherence of 65.4% when subsuming all IC groups (*Fig. 2*). After adjusting for multiple testing, differences were observed between the intermediate and advanced (*P*_adj_ = 0.004) as well as the intermediate and expert groups (*P*_adj_ < 0.001). Interestingly, when the 33 adherence measures were classified according to whether adherence responsibility relied on the patient or on the medical staff, adherence for medical staff was 79.9% and for patients was 38.9% (*P*_adj_ < 0.001; *Fig. 2*). At the same time, complexity did not affect the adherence of the medical staff but did affect the patients’ adherence (*Table 3*, *Fig. 1b*, *c*).

**Table 3:** Adherence for each specific ERAS measure

Adherence category/ERAS measure		Iwate grade				SMD	*P*
		Low (*n* = 17)	Intermediate (*n* = 81)	Advanced (*n* = 74)	Expert (*n* = 71)		
Overall adherence (%), mean(s.d.)		68.9(8.8)	68.4(8.8)	63.6(8.6)	62.9(8.7)	0.432	**<0.001**
Patient-centred adherence* (%), mean(s.d.)		49.0(27.2)	45.3(22.9)	36.2(20.5)	31.9(24.0)	0.420	**0.001**
Medical staff-centred adherence* (%), mean(s.d.)		83.1(7.7)	89.0(7.5)	78.8(7.7)	79.3(7.6)	0.315	0.098
**Preadmission phase (%), mean(s.d.)**		86.3(22.2)	87.2(22.7)	89.0(25.2)	91.5(19.1)	0.246	**0.042**
1	Nutritional status surveyed	100.0 (17/17)	100.0 (81/81)	100.0 (74/74)	100.0 (71/71)	<0.001	1.000
2	Preoperative immunonutrition	100.0 (1/1)	100.0 (7/7)	87.5 (7/8)	100.0 (2/2)	–	0.741
3	Smoking behaviour	0.0 (0/2)	0.0 (0/14)	5.6 (1/18)	12.5 (1/8)	0.316	0.607
4	Alcohol consumption	0.0 (0/3)	10.0 (1/10)	5.9 (1/17)	25.0 (2/8)	0.433	0.483
**Preoperative phase (%), mean(s.d.)**		81.2(15.9)	82.0(11.4)	82.8(12.8)	83.8(14.0)	0.112	0.775
5	Education on the ERAS programme	100.0 (17/17)	100.0 (81/81)	98.6 (73/74)	100.0 (71/71)	0.082	0.516
6	Carb loading	70.6 (12/17)	83.5 (66/79)	90.5 (67/74)	84.3 (59/70)	0.259	0.196
7	Bowel preparation	100.0 (17/17)	98.8 (80/81)	97.3 (72/74)	100.0 (71/71)	0.148	0.494
8	Preoperative sedative medication	68.8 (11/16)	76.5 (62/81)	67.6 (50/74)	67.6 (48/71)	0.103	0.566
9	Antibiotic prophylaxis	100.0 (17/17)	97.5 (79/81)	100.0 (74/74)	100.0 (71/71)	0.112	0.260
10	Thrombosis prophylaxis	52.9 (9/17)	42.0 (34/81)	41.9 (31/74)	53.5 (38/71)	0.152	0.397
11	Steroid administration	70.6 (12/17)	79.0 (64/81)	83.8 (62/74)	77.5 (55/71)	0.163	0.606
**Intraoperative phase (%), mean(s.d.)**		85.9(13.7)	77.0(14.9)	72.6(11.2)	79.0(14.0)	0.647	**<0.001**
12	Type of incision	Measure only applicable for open surgery					
13	Abdominal drains	41.2 (7/17)	29.6 (24/81)	8.1 (6/74)	1.4 (1/71)	0.642	**<0.001**
14	Omentoplasty	–	0.0 (0/20)	21.4 (3/14)	0.0 (0/12)	–	**0.028**
15	PONV prophylaxis	90.9 (10/11)	78.8 (26/33)	86.7 (26/30)	91.7 (33/36)	0.203	0.451
16	Systemic opioid administration	100.0 (17/17)	100.0 (81/81)	100.0 (74/74)	100.0 (71/71)	<0.001	1.000
17	Epidural anaesthesia	Measure only applicable for open surgery					
18	Upper body warming	100.0 (17/17)	100.0 (81/81)	100.0 (74/74)	100.0 (71/71)	<0.001	1.000
19	Use of 0.9 NaCl	100.0 (17/17)	100.0 (81/81)	100.0 (74/74)	100.0 (71/71)	<0.001	1.000
20	Removal of gastric tube	88.2 (15/17)	71.6 (58/81)	63.5 (47/74)	63.4 (45/71)	0.327	0.169
21	Central venous pressure	40.0 (4/10)	79.7 (59/74)	78.1 (57/73)	70.0 (49/70)	0.455	**0.036**
**Postoperative phase (%), mean(s.d.)**		52.4(16.8)	54.8(15.4)	46.7(15.7)	44.2(18.2)	0.376	**0.001**
22	Termination of i.v. fluid administration	94.1 (16/17)	87.7 (71/81)	66.2 (49/74)	57.7 (41/71)	0.549	**<0.001**
23	Postoperative weight gain	70.0 (7/10)	51.9 (27/52)	40.6 (13/32)	48.4 (15/31)	0.308	0.423
24	Energy consumption at POD0	28.6 (4/14)	12.0 (9/75)	11.9 (8/67)	13.4 (9/67)	0.213	0.393
25	Energy consumption at POD1	21.4 (3/14)	24.0 (18/75)	20.9 (14/67)	19.4 (13/67)	0.057	0.398
26	Mobilization at all on POD0	50.0 (8/16)	48.8 (39/80)	18.3 (13/71)	17.9 (12/67)	0.466	**<0.001**
27	Mobilization on POD1	37.5 (6/16)	44.2 (34/77)	30.6 (22/72)	17.9 (12/67)	0.314	**0.009**
28	Mobilization on POD2	42.9 (6/14)	43.4 (33/76)	36.1 (26/72)	24.2 (16/66)	0.226	0.109
29	Mobilization on POD3	77.8 (7/9)	63.8 (44/69)	50.0 (36/72)	38.8 (26/67)	0.457	**0.012**
30	Removal of IUC	100.0 (14/14)	95.7 (67/70)	85.5 (59/69)	80.3 (57/71)	0.425	**0.017**
31	Control postoperative glycaemia	52.9 (9/17)	60.5 (49/81)	78.4 (58/74)	97.2 (69/71)	0.64	**<0.001**
32	Postoperative epidural	Measure only applicable for open surgery					
33	30-day follow-up	94.1 (16/17)	87.7 (71/81)	79.2 (57/72)	81.7 (58/71)	0.238	0.470

Values are % (patient numerator/denominator) unless otherwise stated. In parentheses, the numerator indicates how many patients adhered to the respective ERAS measure, while the denominator indicates for how many patients the respective ERAS measure was applicable and controlled/measured. Kruskal–Wallis test for categorical variables (individual ERAS measures). ANOVA for continuous variables. *Allocation see *Table 1*. ERAS, enhanced recovery after surgery; IUC, indwelling urinary catheter; i.v., intravenous; NaCl, sodium chloride; NRS 2002, Nutritional Risk Screening^19^; POD, postoperative day; PONV, postoperative nausea and vomiting; SMD, standardized mean difference. Statistically significant *P*-values are highlighted in bold.

In the examination of the individual perioperative phases, an increase in adherence was observed in the preadmission phase with increasing complexity of the liver resection (86.3% in the low *versus* 87.2% in the intermediate *versus* 89.0% in the advanced *versus* 91.5% in the expert group, *P* = 0.042; SMD = 0.246), with no significant differences in any of the preadmission measures themselves. Differences occurred between the advanced and expert groups (*P*_adj_ = 0.027). This contrasts with the decline in overall, intraoperative and postoperative adherence with increasing complexity (*Table 3*). The postoperative adherence was the lowest of all perioperative phases and showed the biggest range of adherence between the complexity groups (52.4% in the low *versus* 54.8% in the intermediate *versus* 46.7% in the advanced *versus* 44.2% in the expert group, *P* = 0.001; SMD = 0.376) and, after adjustment, between the intermediate and advanced (*P*_adj_ = 0.014) and the intermediate and expert groups (*P*_adj_ < 0.001).

**Fig. 3:**
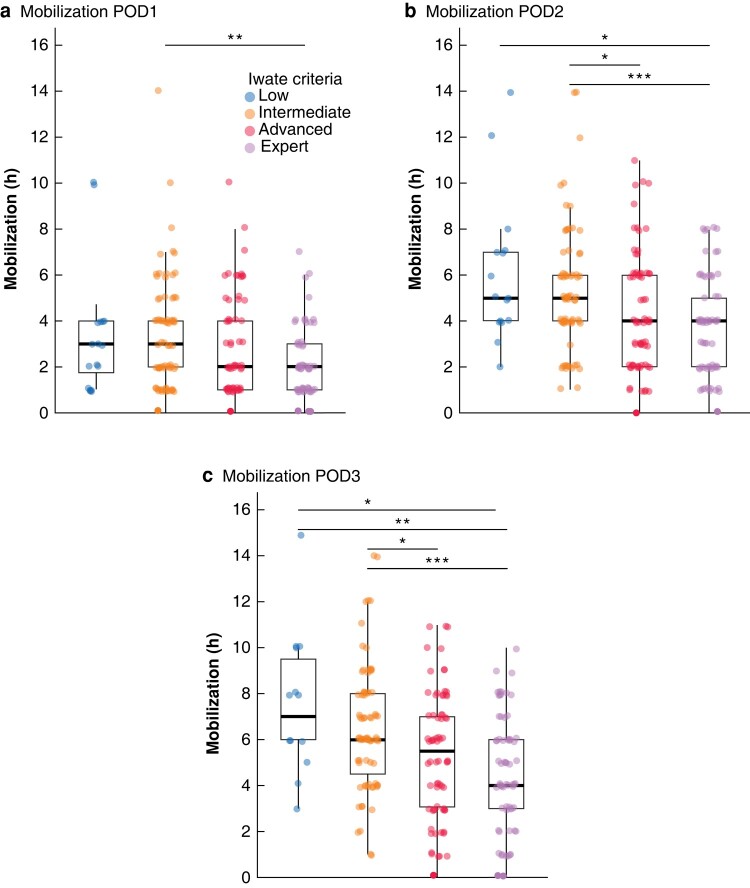
Mobilization (time in hours outside the bed) on postoperative day 1 (a), postoperative day 2 (b), and postoperative day 3 (c) *P_adj_ < 0.05, **P_adj_ < 0.01, ***P_adj_ < 0.001, Welch´s *t*-test with Bonferroni correction. POD, postoperative day.

**Fig. 4:**
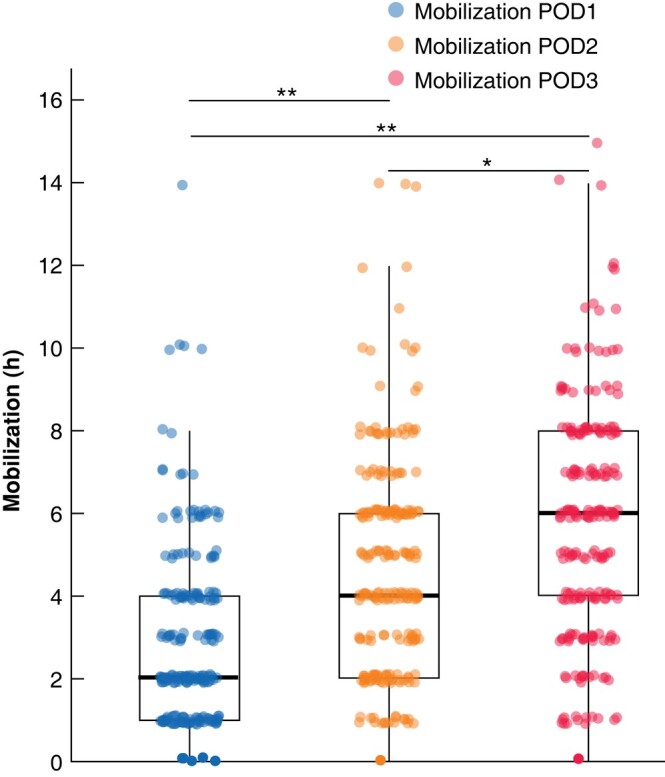
Comparison of mobilization on POD1 *versus* mobilization on POD2 *versus* mobilization on POD3 independent of Iwate criteria *P_adj_ < 0.001, **P_adj_ < 0.0001, Welch´s *t*-test with Bonferroni correction. POD, postoperative day.

The biggest contributors to the differing adherences were the mobilization measures. The rate of patients who mobilized into the stand on the day of surgery was roughly 50% in the low (*n* = 8) and intermediate (*n* = 39) groups and slightly less than 20% in the advanced (*n* = 13) and expert (*n* = 12) groups (*P* < 0.001; SMD = 0.466; *Table 3*, *Fig. S2*). After adjusting for multiple testing, significant differences were found between the low and advanced (*P*_adj_ = 0.048), low and expert (*P*_adj_ = 0.045), intermediate and advanced (*P*_adj_ <0.001) as well as the intermediate and expert groups (*P*_adj_ < 0.001). When analysing the mobilization hours, time decreased with increasing complexity in liver resection regardless of postoperative day, POD (*Table 3*; *Fig. 3a–c*). When grouping liver resections according to IC, there was a significant increase of mobilization time with increasing complexity on POD1, POD2 and POD3 (*Fig. S3*). Overall, mobilization increased from POD1 to POD3 when subsuming all patients (*Fig. 4*).

Another postoperative measure that showed significant decreases in adherence with increasing complexity were the termination of intravenous fluid administration before the second postoperative night (*P* < 0.001; SMD = 0.549) with significant pairwise comparisons between low (*n* = 16) and expert (*n* = 41) groups (94.1% *versus* 57.7% respectively, *P*_adj_ = 0.031), intermediate (*n* = 71) and advanced (*n* = 49) groups (87.7% *versus* 66.2% respectively *P*_adj_ = 0.009) as well as between the intermediate and expert groups (*P*_adj_ < 0.001; *Table 3*; *Fig. S4c*). The removal of an indwelling urinary catheter showed a significant difference between the intermediate (*n* = 67) and expert (*n* = 57) groups (95.7% *versus* 80.3% respectively, *P*_adj_ = 0.031).

Omission of abdominal drains showed significant differences between 41.2% (*n* = 7) in the low and 8.1% (*n* = 6) in the advanced group (*P*_adj_ = 0.003), between the low and expert (1.4%; *n* = 1) groups (*P*_adj_ < 0.001), between the intermediate (29.6%; *n* = 24) and advanced (*P*_adj_ = 0.004) as well as the intermediate and expert groups (*P*_adj_ < 0.001; *Table 3*; *Fig. S4b*).

Adherence to a low central venous pressure during the resection phase showed a significant difference between 40.0% (*n* = 4) in the low and 79.7% (*n* = 59) in the intermediate groups (*P*_adj_ = 0.042; *Fig. S2*).

## Discussion

In this study, adherence to individual ERAS measures was assessed in patients who underwent MILS graded according to the complexity of the liver resection based on the Iwate criteria. The degree of IC is notably directly related to the overall adherence to such a protocol: the easier the liver resection was, the higher the overall adherence rate was. Thus, the needs of complex liver resections for an ERAS protocol and adherence calculation were different from those for easy liver resections and a ‘one-size-fits-all’ approach in liver surgery is more than questionable. Furthermore, adherence was 65%, but was 40% lower when responsibility was with the patient than when responsibility was with the medical staff. Such a high difference between medical staff-centred and patient-centred adherence leads to a new perspective on where improvements of perioperative concepts can be achieved.

The ERAS item with the strongest correlation to the complexity of the liver resection was undoubtedly postoperative mobilization, where adherence showed a significant decrease with rising complexity. Since the implementation of the ERAS protocol at the Charité Virchow–Klinikum, there have been merely incremental enhancements in adherence to mobilization measures. Upon closer examination, it became apparent that the compliance calculations offered by the EAIS presented a distorted view of the observed mobilization progress on the ward: patients are deemed adherent only if they surpass specific thresholds of mobilization hours on POD1, 2 and 3 (≥4 h, ≥6 h, ≥6 h respectively). Patients who, for instance, mobilized for just 4 h on the second postoperative day have achieved two-thirds of the mobilization target but are still categorized as 100% non-adherent (or 0% adherent) to this ERAS measure. Given that the mobilization targets lack evidence specific to liver surgery, the presentation of these liver-specific data could serve as a foundation for potential future RCTs. In the updated ERAS guidelines on liver surgery from 2022 early mobilization was strongly recommended but ‘no recommendation can be made regarding the optimal duration of mobilization’, as data is lacking^4^. This was the first study to report on actual hours mobilized after MILS, but future RCTs are needed to determine whether these complexity-dependent mobilization targets improve outcomes for patients.

An ERAS measure that showed not only strong correlation to the procedure’s complexity but overall low adherence as well was the complete omission of abdominal drainages. This seems to be an everlasting issue during the implementation of an ERAS protocol in liver surgery, especially as even the current guidelines on ERAS in liver surgery cannot give a clear recommendation, but only state that ‘the routine use of abdominal drain placement is not indicated for hepatectomy without biliary reconstruction’^4^. This recommendation is implemented as a strict ‘no-drain’ policy in the EIAS, considering any use of drains as non-adherent, regardless of the complexity of the procedure, but this rule does not reflect the clinical setting^2^. The drain policy at Charité–Universitätsmedizin Berlin during the interval of this study was to avoid it for wedge or smaller resections and for more extensive procedures it may be used at the discretion of the surgeon. If a drain is placed, it will be removed on the second postoperative day if no complications were observed.

An important aspect of assessing adherence was the division of responsibility of implementing ERAS measures between patients and medical staff. Patient-centred ERAS measures generally showed a decreased adherence with rising complexity, while this was not present for medical staff-centred ERAS measures. The measures performed by medical professionals were performed almost independently of the severity of the surgery, it rather concerns all liver surgery patients and the measure as such must be checked for its practicability. On the other hand, the ERAS measures performed by patients may have to be adapted to the severity of the surgery, since the distress of patients differed among procedures. This was evident during the intra- and postoperative phase, as ERAS measures such as early mobilization and omission of drains were more likely to be implemented in minor procedures.

In a comparative trial by Teixeira *et al*. the adherence was 65% (in line with the present study) based on data of 35 patients, of which only 14 patients (40%) underwent MILS^8^. Jones *et al*. reported an overall adherence of 98.2% in the ERAS group (46 patients) after open liver resection, with early removal of the urinary diversion being the only measure not followed by all ERAS patients^9^, and mobilization was defined as ‘twice daily’ without specifying a time range or cut-off values, which makes comparison to the present data difficult. Labgaa *et al*. reported an overall adherence of 73.8% after implementing an ERAS protocol with 22 ERAS measures and, compared with the non-ERAS phase, an increased adherence was seen in 15 measures; main differences were observed in the pre- and postoperative phases^6^. The aforementioned studies included patients who primarily underwent open liver resection, which is nowadays only performed when MILS is technically not possible^20^. Many comparable studies on liver surgery, even those on the ERAS guidelines, incorporate mainly open liver procedures. This skews the reported adherence rates due to the fact that the population and indications for open liver surgery vary drastically from those for MILS^20,21^. Studies including far more complex open procedures may not be representative of most cases in modern liver surgery and it would not be appropriate to create recommendations based on their findings. The aforementioned studies reported adherence using different ERAS protocols independent of the ERAS guidelines for liver surgery, which limited comparability.

For the ERAS-protocol of the EIAS, the 23 ERAS items of the guideline (25 items in the updated guideline) were transformed into 33 measures, which have been applied perioperatively. Dionisios Vrochides *et al*. described the overall adherence to the ERAS protocol of the EIAS database in liver surgery, but their study did not investigate the adherence rates to the individual measures^2,19,22^.

The success of an ERAS protocol always relies on the adherence to it^23^. In a field as complicated and broad as liver surgery, it may be helpful to incorporate the differences in complexity of the procedure into the goals of perioperative management and recovery. The data from this study may help to identify the first liver-specific mobilization thresholds and to apply them in a procedure-dependent manner. Furthermore, patients should not be considered as non-adherent if they have not reached the predefined target and a ‘gradual adherence’ seems far more suitable to reflect the current status of the implementation of the ERAS protocol. As a side-effect not to be neglected, this may lead to greater acceptance by the implementing parties (for example physiotherapists and nursing), who so far receive an adherence of 0% in the EIAS database.

When assessing clinical outcomes within an ERAS protocol, morbidity rate and length of stay are of particular interest. In this study an overall increase in complication rates was seen with an increasing complexity, which falls in line with publications validating the Iwate criteria^12,16,17,24^. Interestingly though, the lowest complication rate occurred within the intermediate IC group and not the low one. This could be explained by the small number of patients in the low IC group. It may be helpful to stratify the cohort based on specific conditions. For instance, cases involving hepatocellular carcinoma accompanied by cirrhosis in individuals aged 70 and above could potentially lead to complications even with a minor resection. Conversely, a younger woman with an adenoma might encounter fewer postoperative complications even following a major resection. Implementing such stratification would naturally result in an even smaller cohort size. Further investigation of such specific constellations was nevertheless not the core subject of this study.

The present analysis also had some limitations. The study was observational and patients were not randomized according to the complexity of the liver resection. The unravelled findings are impacted by possible surrogate parameters reflected by an increasing rate of malignant lesions between the groups as well as neoadjuvant chemotherapy rate with increasing degree of complexity. For all other preoperative parameters, there were no clinically relevant differences between the groups. Only patients who underwent MILS were included in the study. Open liver resections such as complex procedures with hepatobiliary reconstructions were excluded. Hence, the present data cannot be generalized to all liver resections. IC were not developed for open liver resections. This means that the graduation of complexity of open liver resections is probably not analogous to the graduation performed in this study.

The present data indicated that adherence decreases with increasing severity of MILS. A one-size-fits-all approach could be questionable and thresholds need to be individualized for patient adherence.

The greatest potential for improvement in an ERAS programme is on the patient-centred adherence side, as this was significantly lower than medical staff-centred adherence. This insight leads to a new perspective on where improvements can be made in clinical practice.

## Supplementary Material

zrad147_Supplementary_Data

## Data Availability

Data is not publicly available, as sharing the data is not covered by the ethical approval.
