# Supplementary material for: Impact of complexity in minimally invasive liver surgery on enhanced recovery measures: prospective study
Source: BJS Open. 2024 Jan 19;8(1):zrad147. doi: 10.1093/bjsopen/zrad147 (PMC10799324; doi:10.1093/bjsopen/zrad147)
Supplement: zrad147_Supplementary_Data [file zrad147_supplementary_data.docx]

**The impact of complexity in minimally invasive liver surgery on enhanced recovery measures: prospective study**

Paul M Dahlke^1^, Christian Benzing^1^, Georg Lurje^1^, Thomas Malinka^1^, Nathanael Raschzok^1,2^, Can Kamali^1^, Safak Gül-Klein^1^, Wenzel Schöning^1^, Karl H Hillebrandt^1,2^, Johann Pratschke^1^, Jens Neudecker^1^, Felix Krenzien^1,2^

^1^Department of Surgery, Campus Charité Mitte and Campus Virchow-Klinikum, Charité – Universitätsmedizin, corporate member of Freie Universität Berlin, Humboldt-Universität zu Berlin, and Berlin Institute of Health, Berlin, Germany

^2^Berlin Institute of Health (BIH), 10178 Berlin, Germany

**Corresponding author:**

PD Dr. med. Felix Krenzien

Department of Surgery,

Campus Charité Mitte and Campus Virchow-Klinikum,

Charité Universitätsmedizin Berlin,

Augustenburger Platz 1,

13353 Berlin, Germany.

E-mail: felix.krenzien@charite.de

Phone: (+49) 30450552001

**Supplementary Materials - Index**

| **Supplementary Methods** |  |
| --- | --- |
| None | *pag. 3* |
| **Supplementary Results** |  |
| None | *pag. 4* |
| **Supplementary Appendixes** |  |
| STROBE Statement | *pag. 5* |
| **Supplementary Figures and Tables** |  |
| Table S1 | *pag. 8* |
| Figure S1 | *pag. 10* |
| Figure S2 | *pag. 10* |
| Figure S3 | *pag. 11* |
| Figure S4 | *pag. 11* |
| **References** | *pag. 12* |

**Supplementary Methods**

- None

**Supplementary Results**

- None

**Supplementary Appendixes**

- STROBE Statement

| **Section/item** | | **Nr.** | **Recommendation** | **Line** |
| --- | --- | --- | --- | --- |
| **Title and Abstract** | | 1 | (a) Indicate the study’s design with a commonly used term in the title or the abstract | 38 |
|  |  |  | (b) Provide in the abstract an informative and balanced summary of what was done and what was found | 33-53 |
|  |  |  |  |  |
| **Introduction** | | | |  |
|  | Background/ rationale | 2 | Explain the scientific background and rationale for the investigation being reported | 56-85 |
|  |  |  |  |  |
|  | Objectives | 3 | State specific objectives, including any prespecified hypotheses | 87-88 |
|  |  |  |  |  |
| **Methods** | |  |  |  |
|  | Study design | 4 | Present key elements of study design early in the paper | 92-100 |
|  |  |  |  |  |
|  | Setting | 5 | Describe the setting, locations, and relevant dates, including periods of recruitment, exposure, follow-up, and data collection | 95-100 |
|  |  |  |  |  |
|  | Participants | 6 | (a) ***Cohort study***—Give the eligibility criteria, and the sources and methods of selection of participants. Describe methods of follow-up  ***Case-control study***—Give the eligibility criteria, and the sources and methods of case ascertainment and control selection. Give the rationale for the choice of cases and controls  ***Cross-sectional study***—Give the eligibility criteria, and the sources and methods of selection of participants | 102-111 |
|  |  |  | (b) ***Cohort study***—For matched studies, give matching criteria and number of exposed and unexposed Case-control study—For matched studies, give matching criteria and the number of controls per case | N/A |
|  |  |  |  |  |
|  | Variables | 7 | Clearly define all outcomes, exposures, predictors, potential confounders, and effect modifiers. Give diagnostic criteria, if applicable | 120-135 |
|  |  |  |  |  |
|  | Data sources/ measurement | 8 | For each variable of interest, give sources of data and details of methods of assessment (measurement). Describe comparability of assessment methods if there is more than one group | 120-135 |
|  |  |  |  |  |
|  | Bias | 9 | Describe any efforts to address potential sources of bias | N/A |
|  |  |  |  |  |
|  | Study size | 10 | Explain how the study size was arrived at | Figure S1 |
|  |  |  |  |  |
|  | Quantitative variables | 11 | Explain how quantitative variables were handled in the analyses. If applicable, describe which groupings were chosen and why | 136 -144 |
|  |  |  |  |  |
|  | Statistical methods | 12 | (a) Describe all statistical methods, including those used to control for confounding | 136 -144 |
|  |  |  | (b) Describe any methods used to examine subgroups and interactions | 136 -144 |
|  |  |  | (c) Explain how missing data were addressed | N/A |
|  |  |  | (d) ***Cohort study***—If applicable, explain how loss to follow-up was addressed ***Case-control study***—If applicable, explain how matching of cases and controls was addressed ***Cross-sectional study***—If applicable, describe analytical methods taking account of sampling strategy | N/A |
|  |  |  | (e) Describe any sensitivity analyses | N/A |
|  |  |  |  |  |
| **Results** | |  |  |  |
|  | Participants | 13 | (a) Report numbers of individuals at each stage of study—eg numbers potentially eligible, examined for eligibility, confirmed eligible, included in the study, completing follow-up, and analysed | 154-157 |
|  |  |  | (b) Give reasons for non-participation at each stage | Figure S1 |
|  |  |  | (c) Consider use of a flow diagram | Figure S1 |
|  |  |  |  |  |
|  | Descriptive data | 14 | (a) Give characteristics of the study participants (eg demographic, clinical, social) and information on exposures and potential confounders | 160-177, Table 2 |
|  |  |  | (b) Indicate number of participants with missing data for each variable of interest | N/A |
|  |  |  | (c) ***Cohort study***—Summarise follow-up time (eg, average and total amount) | N/A |
|  |  |  |  |  |
|  | Outcome data | 15 | ***Cohort study***—Report numbers of outcome events or summary measures over time ***Case-control study***—Report numbers in each exposure category, or summary measures of exposure ***Cross-sectional study***—Report numbers of outcome events or summary measures | 180-224 |
|  |  |  |  |  |
|  | Main results | 16 | (a) Give unadjusted estimates and, if applicable, confounder-adjusted estimates and their precision (eg, 95% confidence interval). Make clear which confounders were adjusted for and why they were included | 180-215 |
|  |  |  | (b) Report category boundaries when continuous variables were categorized | 134-135 |
|  |  |  | (c) If relevant, consider translating estimates of relative risk into absolute risk for a meaningful time period | N/A |
|  |  |  |  |  |
|  | Other analyses | 17 | Report other analyses done—eg analyses of subgroups and interactions, and sensitivity analyses | 216-224 |
|  |  |  |  |  |
| **Discussion** | |  |  |  |
|  | Key results | 18 | Summarise key results with reference to study objectives | 227-286 |
|  |  |  |  |  |
|  | Limitations | 19 | Discuss limitations of the study, taking into account sources of potential bias or imprecision. Discuss both direction and magnitude of any potential bias | 310-318 |
|  |  |  |  |  |
|  | Interpretation | 20 | Give a cautious overall interpretation of results considering objectives, limitations, multiplicity of analyses, results from similar studies, and other relevant evidence | 269-308 |
|  |  |  |  |  |
|  | Generalisability | 21 | Discuss the generalisability (external validity) of the study results | 288-308 |
|  |  |  |  |  |
| **Other information** | | |  |  |
|  | Funding | 22 | Give the source of funding and the role of the funders for the present study and, if applicable, for the original study on which the present article is based | 329 |
|  |  |  |  |  |
| **Note:** An Explanation and Elaboration article discusses each checklist item and gives methodological background and published examples of transparent reporting. The STROBE checklist is best used in conjunction with this article (freely available on the Web sites of PLoS Medicine at http://www.plosmedicine.org/, Annals of Internal Medicine at http://www. annals.org/, and Epidemiology at http://www.epidem.com/). Information on the STROBE Initiative is available at www.strobe-statement.org. | | | | |

**Supplementary Figures and Tables**

| **Table S1** | | **ERAS protocol and adherence conditions.** Patients were considered adherent to the respective ERAS measure if this ERAS measure was applicable to them and the corresponding adherence conditions of the EIAS database were met. Previously published by Schmelzle et al. (1) | |
| --- | --- | --- | --- |
| **ERAS-Measure / ERAS-Item ^a^** | | | **Adherence** (applicable when) |
| **Pre-admission** | | |  |
| **1 (2)** | Nutritional status  surveyed | | Nutritional status was surveyed. |
| **2 (3)** | Preoperative | | If nutritional status is risky (NRS 2002 ≥ 3 points), administration of preoperative immunonutrition. |
| **3 (-)** | Smoking behaviour | | Smoking was stopped at least four weeks prior to surgery. Not applicable for non-smokers |
| **4 (-)** | Alcohol consumption | | Any alcohol consumption stopped at least four weeks before surgery. Not applicable for non-users. |
|  |  | |  |
| **Preoperatively** | | |  |
| **5 (1)** | Education on the ERAS program | | Patients were educated on the ERAS program pre-operatively. |
| **6 (4)** | Carbloading | | A high-calorie drink was taken on the evening before the operation and 2 h before the operation. Not applicable in patients with DM. |
| **7 (5)** | Bowel preparation | | No bowel preparation (preoperative laxative measures) was performed. |
| **8 (6)** | Preoperative sedative  medication | | No long-acting sedative medication was administered preoperatively. |
| **9 (9)** | Antibiotic prophylaxis | | An i.v. antibiotic was administered within the last hour before surgery |
| **10 (7)** | Thrombosis prophylaxis | | LMWH was administered perioperatively. Also adherent if this was contraindicated and therefore not administered. |
| **11 (8)** | Steroid administration | | Patients with DM have not been administered steroids perioperatively. Patients without DM have been administered steroids perioperatively. |
|  | | |  |
| **Intraoperatively** | | |  |
| **12 (10)** | Type of incision | | No Mercedes-type incision has been selected for open procedures. Not applicable for minimally invasive procedures. |
| **13 (13)** | Abdominal drains | | No abdominal drains were placed. |
| **14 (17)** | Omentoplasty | | In case of (extended) hemihepatectomy on the left, an omentoplasty was applied to the resection surface. |
| **15 (21)** | PONV prophylaxis | | When at risk for PONV (Apfel score (2) ≥ 2), multimodal PONV prophylaxis (drug and nondrug) was administered. |
| **16 (20)** | Systemic opioid administration | | No or only short-acting opioids (fentanyl, remifentanil, sufentanil) were administered intraoperatively. |
| **17 (20)** | Epidural anaesthesia | | PDK anaesthesia has been omitted for open procedures. Not applicable for other minimally invasive procedures. |
| **18 (14)** | Upper body warming | | Convective air heating systems were used. |
| **19 (22)** | Use of 0.9% NaCl | | No 0.9% NaCl solution was infused. |
| **20 (12)** | Removal of gastric tube | | A placed nasogastric tube with procedure end was removed at the end of the operation. |
| **21 (22)** | Central venous pressure | | The CVD immediately before parenchymal transection was <5 mmHg. |
|  |  | |  |
| **Postoperatively** | | |  |
| **22 (22)** | Termination of i.v. fluid administration | | i.v. fluid delivery was stopped after ≤1 night. |
| **23 (22)** | Postoperative weight gain | | The weight gain from pre- to postoperative was a maximum of 2 kg. Thus, fluid balance was verified. |
| **24 (15)** | Energy consumption at POD0 | | On the day of surgery postoperatively, ≥ 300 kcal were consumed via sip feeds. |
| **25 (15)** | Energy consumption at POD1 | | On day 1 postoperatively, ≥ 600 kcal were consumed via sip feeds. |
| **26 (19)** | Mobilisation on the day of surgery | | On the day of surgery postoperatively, any mobilization took place outside the bed. |
| **27 (19)** | Mobilisation on POD1 | | On POD1, ≥4 h of mobilization took place outside the bed. |
| **28 (19)** | Mobilisation on POD2 | | On POD2, ≥6 h of mobilization took place outside the bed. |
| **29 (19)** | Mobilisation on POD3 | | On POD3, ≥6 h of mobilization took place outside the bed. |
| **30 (-)** | Removal of IUC | | IUC was removed after one night at the latest. |
| **31 (16)** | Control postoperative glycemia | | Blood glucose levels were controlled postoperatively. |
| **32 (20)** | Postoperative epidural | | Epidural anaesthesia was also dispensed with postoperatively for open procedures. Not applicable for other minimally invasive procedures. |
| **33 (-)** | 30 days follow-up | | Patients were contacted 30 days after surgery to document any post-inpatient complications. |
|  |  | |  |
| **IUC**, indwelling urinary catheter; **DM**, diabetes mellitus; **i.v.**, intravenous; **LMWH**, low-molecular-weight heparin; **NaCl**, sodium chloride; **NRS 2002**, Nutritional Risk Screening (3); **POD**, postoperative day; **PONV**, postoperative nausea and vomiting; **CVD**, central venous pressure.  **^a^** first number represents the measure applied in this ERAS protocol (33 measure), number in parentheses represents the corresponding guideline items (23 items; empty if no corresponding guideline item exists). | | | |

***Figure S1:*** *Patient inclusion and exclusion criteria and flowchart.*

***
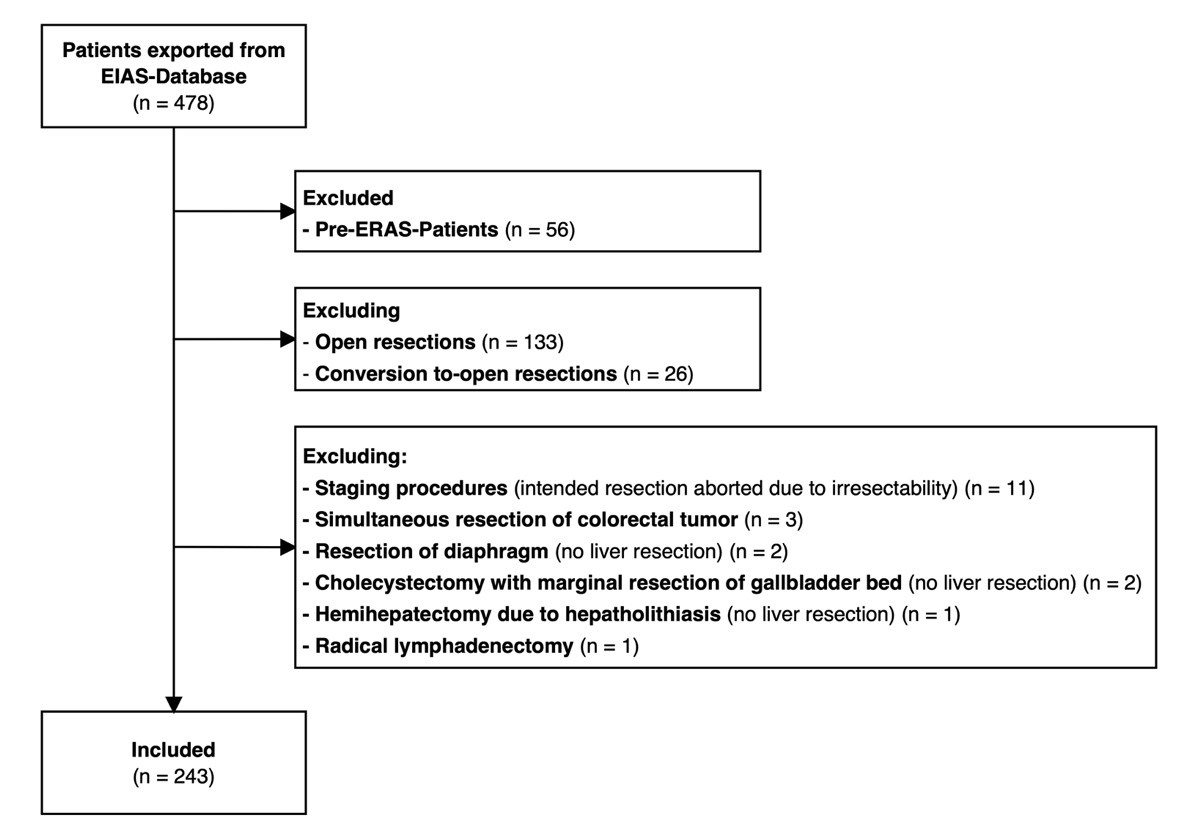
***

***Figure S2:*** *The levels of perioperative adherence to each ERAS measure (n = 30) were grouped by Iwate criteria. To display the results, a heat map was compiled by Wards’ minimum variance hierarchical clustering with Euclidean distance.* ***ERAS****, Enhanced Recovery After Surgery;* ***ASA****, American Society of Anesthesiologists;* ***POD****, postoperative day;* ***CVP****, central venous pressure.*

**
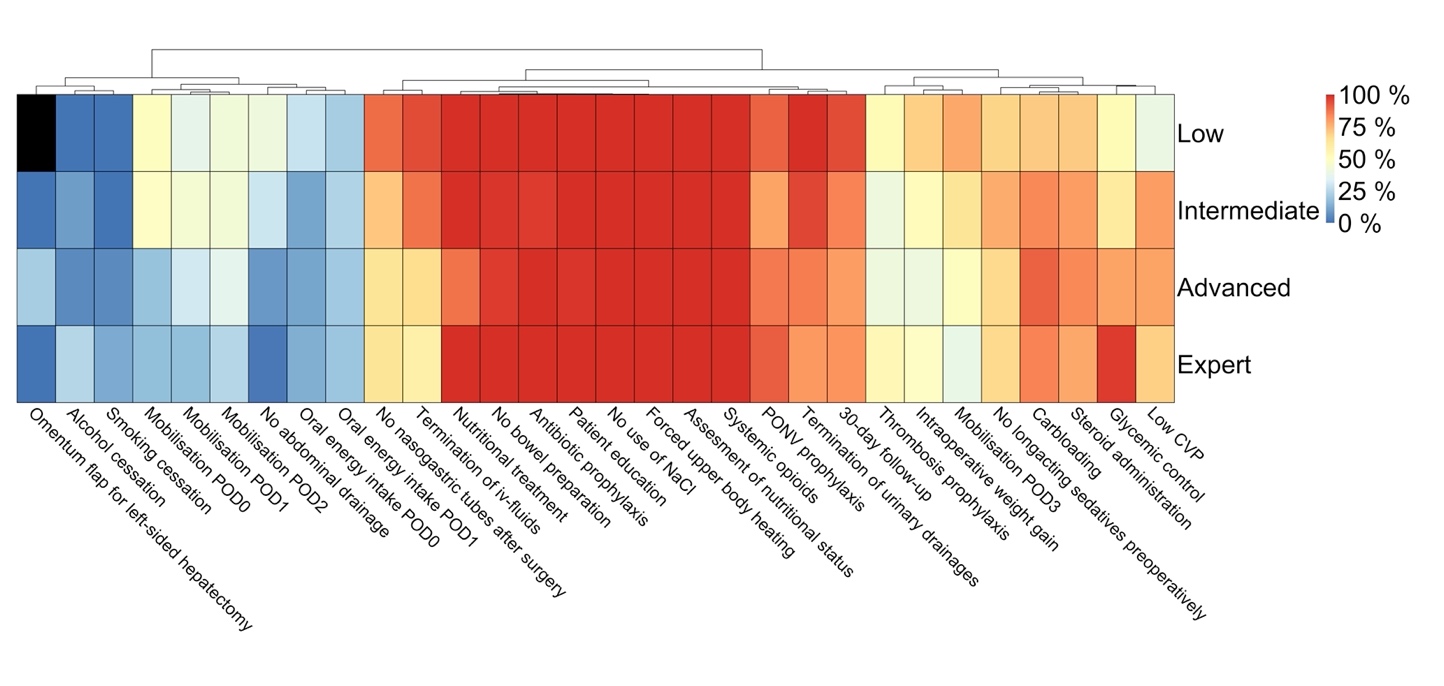
**

***Figure S3:*** *Progression of postoperative mobilization (time in hr) by Iwate criteria low (A), intermediate (B), advanced (C) and expert (D).* ***POD****, postoperative day.
(*p<0.05, **p<0.01, ***p<0.001, ****p<0.0001 , Welch´ t-test with Bonferroni correction).*

**
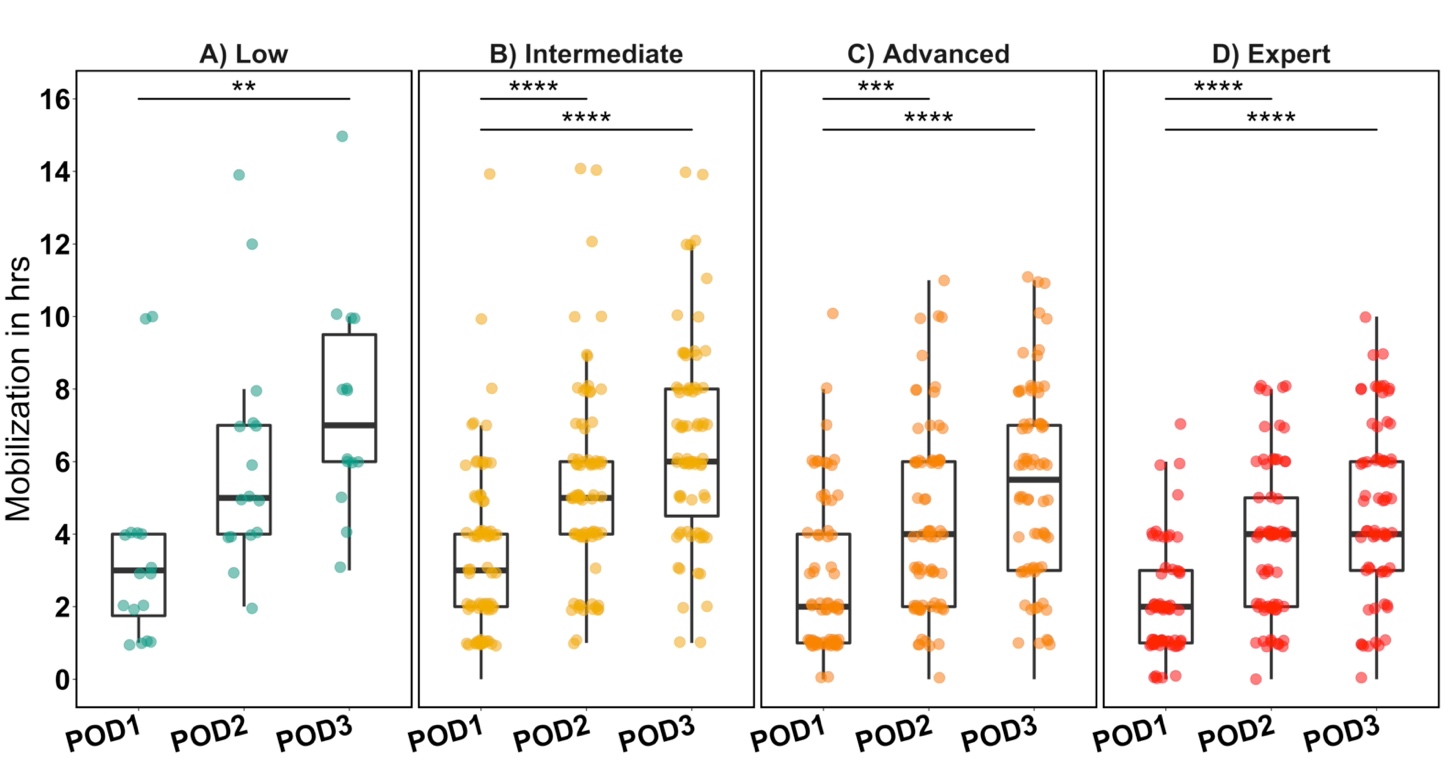
**

***Figure S4:*** *No use of nasogastric tubes after surgery (A), no use of abdominal drainages (B), termination of i.v. fluids within one night after surgery (C).*

*(*P_adj_ <0.05, **P_adj_ <0.01, ***P_adj_ <0.001, **** P_adj_ <0.0001, Wilcoxon rank sum test with Bonferroni correction).*

**
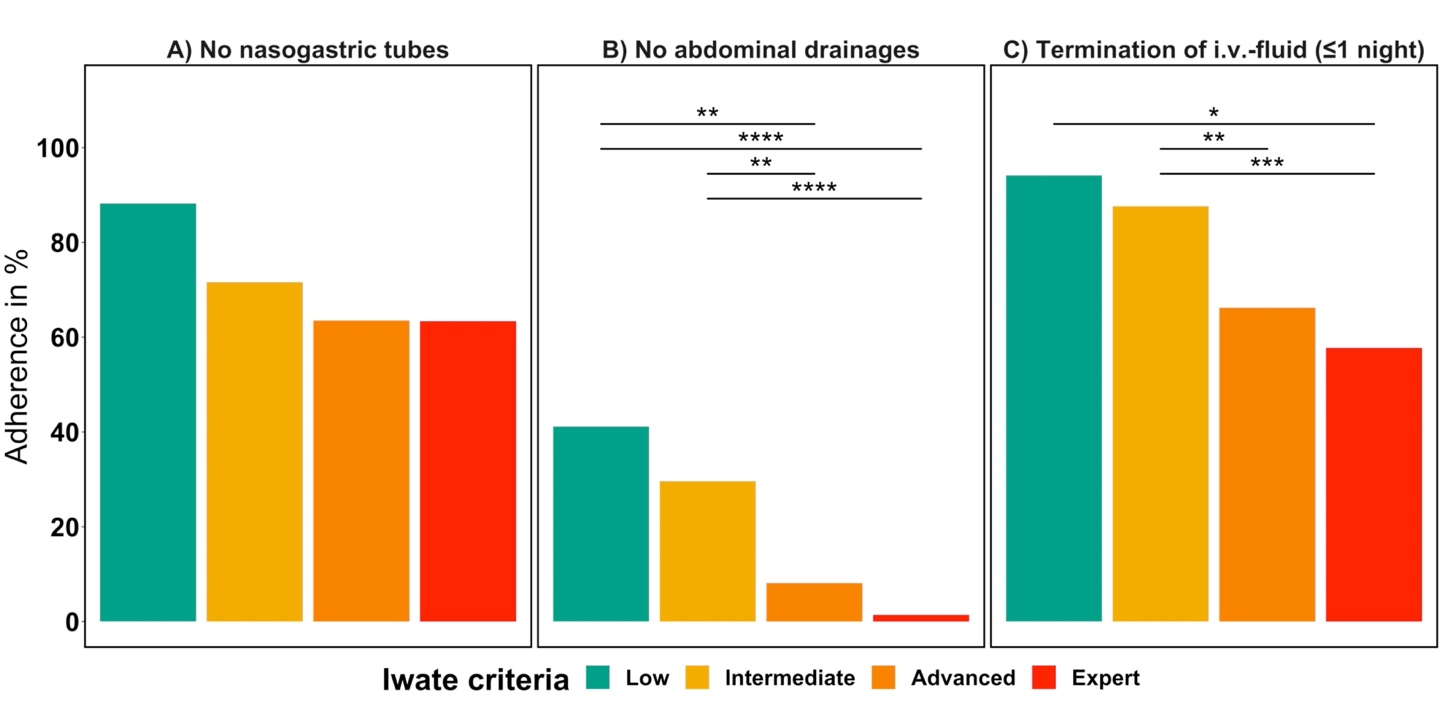
**

**References**

[1 Schmelzle M, Krenzien F, Dahlke P, Krombholz A, Nevermann N, Feldbrügge L, *et al.* Validation of the Enhanced Recovery after Surgery (ERAS) society recommendations for liver surgery: a prospective, observational study. *Hepatobiliary Surg Nutr*. 2021 Jan; **0**: 0–0.](https://sciwheel.com/work/bibliography/12541640)

[2 Apfel CC, Läärä E, Koivuranta M, Greim CA, Roewer N. A simplified risk score for predicting postoperative nausea and vomiting: conclusions from cross-validations between two centers. *Anesthesiology*. 1999 Sep; **91**: 693–700.](https://sciwheel.com/work/bibliography/12031050)

[3 Kondrup J, Allison SP, Elia M, Vellas B, Plauth M, Educational and Clinical Practice Committee, European Society of Parenteral and Enteral Nutrition (ESPEN). ESPEN guidelines for nutrition screening 2002. *Clin Nutr*. 2003 Aug; **22**: 415–421.](https://sciwheel.com/work/bibliography/2425932)
